# Radiological frontiers in understanding paraspinal muscle pathophysiology in chronic low back pain

**DOI:** 10.3389/fmed.2025.1653876

**Published:** 2025-09-23

**Authors:** Xuerou Li, Fuwen Dong, Xiaofei Chen, Xingxin Luo, Wenqi Wang

**Affiliations:** ^1^The First Clinical Medical College of Gansu University of Chinese Medicine, Lanzhou, Gansu, China; ^2^Department of Radiology, Gansu Provincial Hospital of Traditional Chinese Medicine (The first affiliated hospital of Gansu University of Traditional Chinese Medicine), Lanzhou, Gansu, China

**Keywords:** paraspinal muscles, chronic low back pain, imaging techniques, functional MRI, quantitative MRI, fiber-type composition, depth-specific analysis, muscle degeneration

## Abstract

**Background:**

Paraspinal muscles have a profound role in maintaining spinal stability and are often implicated in spinal degenerative conditions as well as chronic low back pain (CLBP). Alterations in these muscles have significant clinical implications for early prevention, treatment strategies, prognosis, and understanding the underlying mechanisms of CLBP. Recent advances in imaging techniques can generate prominent structural and functional characteristics of these muscles.

**Objectives:**

This study is specifically to review recent advancements in imaging techniques focusing on the regenerative and degenerative properties pertinent to paraspinal muscles in the context of CLBP.

**Methods:**

A literature review was executed to ascertain the databases including PubMed, Google Scholar, RelMed, and the National Library of Medicine. The search included studies elucidating recent imaging advancements, fiber-type composition analysis, level/depth-specific muscle characteristics, and clinical applications of novel radiological techniques in evaluating paraspinal muscle morphology and function. We performed this review without comprehensive meta-analysis.

**Results:**

The review identified significant advancements in imaging modalities for assessing paraspinal muscles, including functional MRI (fMRI), quantitative MRI (qMRI), and T2 mapping techniques. Key findings include: Fiber-type composition analysis: Recent studies elucidate the role of depth-dependent fiber-type gradients along with their correlation with muscle function in health and disease. Standardized imaging protocols: The lack of uniform imaging protocols remains a challenge, emphasizing the need for standardization to improve reproducibility and reliability. Radiological advances: Emerging techniques such as advanced fMRI and qMRI enable detailed visualization of muscle structure and function, overcoming limitations of traditional imaging methods. Age-related microvascular changes: age-related microvascular alterations significantly impact paraspinal muscle morphology and can be effectively captured by modern imaging biomarkers.

**Conclusion:**

Advances in imaging techniques have enhanced our understanding of the structural and functional changes in paraspinal muscles associated with CLBP. The integration of imaging biomarkers into clinical practice holds promise for early diagnosis, targeted interventions, and better prognostic evaluations. Future research should focus on developing standardized imaging protocols and further exploring depth-specific properties of paraspinal muscles to enhance clinical outcomes.

## Graphical Abstract

**Figure:**
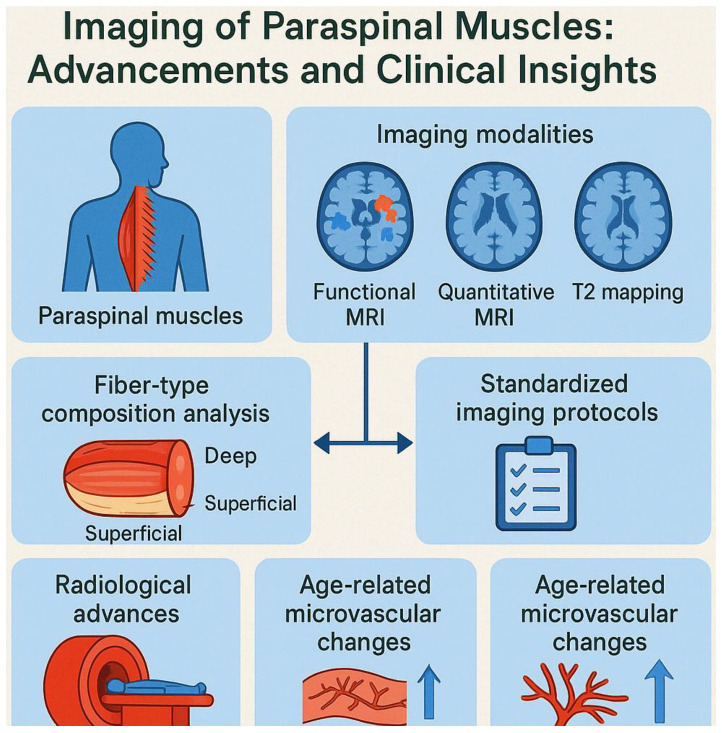

## Introduction

Chronic low back pain (CLBP) is a main contributor to years lived with disability, significantly affecting populations across various age groups (1). Increasing incidence of CLBP in both aging population and young adults has been well-documented, with epidemiological data indicating a yearly prevalence rate of approximately 42.4% in young adults (2). Mechanical characteristics of muscles such as tone as well as stiffness, are integral to ensure optimal muscle function and energy-efficient contractions (3). Lumbar paraspinal musculature,

encompassing the multifidus, erector spinae, psoas major, quadratus lumborum, and other smaller stabilizing muscles exhibits significant implications in spinal movement and stability. Multifidus, located within the spinotransverse group, is particularly crucial for spinal stabilization, while the erector spinae fosters vertebral column extension (4, 5). Psoas major contributes to hip flexion as well as lumbar stabilization (4–7). Alterations across structure and function of these muscles are typically influenced through factors such as age, activity level, and pathology, which are closely linked to spinal health. Notably, peak muscle mass and strength are achieved during the third decade of life, followed by a gradual decline, although significant reductions often occur later in life under healthy conditions (5, 8–13). Disruptions in these muscle properties, particularly in the lumbar myofascial region, have been frequently associated with CLBP, suggesting a potential link to underlying pathophysiological mechanisms (14–16).

Therapeutic interventions, such as manual therapy and targeted exercise programs, are widely implicated for managing CLBP. These modalities have been described the efficacious in modulating paraspinal muscle activity (17–19), thereby further modulating muscle tone and stiffness. Clinicians often rely on palpatory assessments of muscle tone and spinal stiffness (20) to guide therapeutic approaches and monitor outcomes (21). However, manual techniques are frequently criticized for their lack of reliability and consistency (22, 23). Advanced imaging modalities, including ultrasound method of diagnosis (24), magnetic resonance elastography (25), shear wave elastography (26), as well as electromyography, offer enhanced precision in ascertaining muscle properties but are often impractical for routine clinical use due to their high cost, complexity, and operational demands. This creates an ongoing challenge in quantifying paraspinal muscle tone and stiffness in a standard clinical setting (27).

Imaging techniques include computed tomography (CT), ultrasound (US), and MRI offer valuable insights into the structural and functional attributes of paraspinal muscles. However, their routine use in clinical practice is limited by the lack of standardized and easily applicable measures of muscle degeneration. Establishing reliable imaging biomarkers for paraspinal muscle evaluation could pave the way for more robust research linking muscle degeneration to spinal pathologies and LBP (28). Hence, research should prioritize the refinement and validation of novel imaging techniques that are both practical and clinically feasible. Advances in imaging technology typically portable ultrasound devices and machine learning algorithms for image analysis may facilitate efficient elucidation of information related to altered changes in paraspinal muscle morphological features as well as their function. These kinds of emerging innovations could enhance a profound understanding pertinent to the paraspinal muscle function, spinal degenerative alterations, and CLBP, which finally enable development of efficient therapeutic modalities (29).

This review aims to explore the latest advancements related to imaging modalities which could assess both regeneration and degeneration changes in paraspinal muscles in particularly during disease conditions of CLBP. Furthermore, by exploring state-of-the-art imaging techniques, this study also explores the efficient understanding of how paraspinal muscle alterations contribute to the onset, progression, and persistence of CLBP. The application of advanced imaging methods in ascertaining these muscles is essential to identify subtle structural and functional alterations that could lead to more precise advanced imaging methods for diagnosis and personalized therapeutic strategies for CLBP management.

## Literature search and study selection/evaluation

A comprehensive literature search was performed across multiple electronic databases, including National Library of Medicine (NLM), PubMed, Medline, Google Scholar, eMedicine, and Relemed, to identify publications relevant to imaging advances in paraspinal muscles and their role in CLBP. The search strategy combined Medical Subject Headings (MeSH) and free-text keywords such as “*paraspinal muscles*,” “*multifidus*,” “*quadratus lumborum*,” “*chronic low back pain*,” “*functional MRI*,” “*quantitative MRI*,” “*muscle degeneration*,” “*fatty infiltration*,” and “*muscle cross-sectional area (CSA)*.” Boolean operators (AND/OR) were used to refine searches.

### Selection and evaluation

The initial search results were screened by titles and abstracts to exclude irrelevant publications, conference abstracts without full texts, and non-English papers. Full-text evaluation was then conducted for studies that addressed (a) imaging modalities used to evaluate paraspinal muscle structure or function, (b) clinical or biomechanical significance of paraspinal muscle alterations in CLBP, and (c) methodological advancements in quantitative assessment. Both clinical and experimental studies were included, with priority given to publications from the past ≥ 15 years, and the search was conducted up to 30 June 2025 to capture the latest imaging innovations.

### Data extraction and synthesis

Key information including study design, population characteristics, imaging techniques (e.g., MRI, fMRI, qMRI, T2 mapping, ultrasound elastography), outcome measures (CSA, fat fraction, muscle volume, fiber-type distribution), and main findings were extracted. Studies were synthesized narratively and comprehensively to highlight emerging imaging techniques, depth- and side-specific muscle variations, and clinical applications. Where possible, findings from multiple studies were compared to identify consistent patterns, gaps in knowledge, and areas requiring standardization. This integrative synthesis allowed us to critically evaluate current evidence, identify clinically relevant imaging biomarkers, and propose future directions for research and clinical practice.

## Results

The review identified 203 studies in total, comprising 106 observational studies and 10 clinical trials, published till 30 June 2025. These studies formed the basis for the subsequent synthesis and analysis, providing a transparent overview of the scope and coverage of the review.

### Advances in imaging techniques for evaluating lumbar paraspinal muscles in CLBP

#### Imaging techniques for paraspinal muscles

Studies of paraspinal muscle morphology rely primarily on imaging modalities like CT, MRI, and US. Advanced techniques, including MR spectroscopy, chemical shift imaging, and multiecho MRI, have further enabled the analysis of fatty degeneration in the lumbar multifidus (30–33).

#### CT scans and paraspinal muscles

CT imaging is a valuable non-invasive method for assessing muscle density, CSA, and fatty infiltration. Two key indicators of muscle degeneration detected via CT are reduced muscle size and increased fat deposition. Additionally, CT allows for muscle density quantification through Hounsfield Units (HU) (34). Previous studies (28, 35) confirmed CT’s reliability for measuring muscle CSA and density in patients with CLBP.

#### MRI and paraspinal muscles

MRI offers high-resolution images that provide insights into muscle CSA as well as fatty infiltration. It is commonly utilized in the diagnosis of spinal conditions such as tumors, infections, fractures, and disk protrusions. MRI variables related to muscle morphology demonstrate high intraobserver reliability and moderate interobserver agreement (36, 37).

#### MR spectroscopy and multiecho MRI

MR spectroscopy has proven to be a robust method for detecting metabolic changes in lumbar musculature, with findings correlating closely with histological analyses (38). Fischer et al. (31) introduced multiecho MRI as an innovative technique to quantify fat content in the lumbar multifidus, with results aligning with those of MR spectroscopy.

#### Ultrasound imaging

Ultrasound imaging is a reliable and repeatable technique for evaluating muscle size, particularly in the lumbar multifidus. Studies have validated its accuracy for detecting muscle atrophy in LBP patients and healthy populations (39–42). Moreover, Cuellar et al. (43) highlighted that muscle measurements in older adults, using CT, MRI, and US, can achieve moderate to substantial reliability. Several imaging methods, such as CT, MRI, and US, are profound tools to ascertain CSA, density, as well as fatty infiltration of paraspinal muscles (Table 1) (29–43).

**TABLE 1:** This table summarizes various imaging techniques used to study paraspinal muscles and their relevance in chronic low back pain (CLBP).

Imaging technique	Current implications in CLBP	Advantages in paraspinal muscle imaging	References
Computed tomography (CT)	Reliable for assessing muscle density, cross-sectional area (CSA), and fatty infiltration. Detects muscle degeneration indicators like reduced size and increased fat deposition.	Allows muscle density quanti fication using Hounsfield Units (HU). Offers excellent reliability in measuring muscle CSA and density.	(28, 34, 35)
Magnetic resonance imaging (MRI)	High-resolution imaging for diagnosing spinal conditions (tumors, infections, fractures, disk protrusions) and analyzing CSA and fatty infiltration.	Gold standard for detailed assessment of CSA, muscle composition, and morphological features. Demonstrates high intraobserver reliability.	(7, 36, 37, 44–46)
MR spectroscopy	Robust technique for detecting metabolic changes in lumbar musculature, correlating with histological findings.	Offers precise quantification of fat content in muscles, with findings validated against histological analyses.	(31, 38)
Multiecho MRI	Innovative method for fat quantification in lumbar multifidus, with results aligning with MR spectroscopy.	Enhances fat content analysis and complements other MRI techniques for comprehensive muscle evaluation.	(7, 31, 47, 48)
Ultrasound (US)	Accurate and repeatable for measuring muscle size and detecting atrophy in LBP patients and healthy populations.	Cost-effective and portable. Validated for detecting muscle atrophy and assessing CSA and fatty infiltration.	(29–43)

The “imaging technique” column identifies the method utilized, while “current implications in CLBP” highlights its role in assessing muscle morphology and pathology. The “advantages in paraspinal muscle imaging” column details the specific strengths of each technique in evaluating muscle characteristics, including cross-sectional area (CSA), fatty infiltration, and muscle density.

#### Level-specific imaging insights in CLBP

First, the lumbar multifidus should be the primary focus. Across reviews and cohort work, LM shows consistent associations with CLBP reduced CSA, fatty infiltration (FI), and degraded “muscle quality” predict pain intensity and outcomes independent of spinal degeneration (49–52). Quantitative MRI studies and meta-analyses report higher lumbar multifidus fat fraction in symptomatic patients, with FI tracking worse pain and prognosis; several groups also show CSA/FI changes that are predictive up to 12 months. These findings position lumbar multifidus as the sentinel paraspinal for phenotyping CLBP, with erector spinae commonly co-affected and worth secondary analysis (49–52).

Second, quadratus lumborum (QL) deserves explicit coverage. While historical imaging literature is mixed, contemporary pain-medicine and elastography data point to QL involvement via myofascial trigger points, enthesopathy, and altered stiffness patterns in people with LBP (53–55). Clinically, QL pain can refer to the sacroiliac region, buttock, greater trochanter, groin, and even mimic radicular distributions; ultrasound-guided diagnostic blocks/injections are reported as helpful when QL is suspected (53–55). Emerging shear-wave elastography studies quantify increased QL stiffness linked to LBP phenotypes, suggesting a structural-functional correlate that traditional static MRI may miss. Lumbar multifidus remains the primary muscle of interest; erector spinae and psoas major (PM) adjunct assessment; and QL should be actively screened especially in myofascial or movement-provoked LBP (53–55). Third, recommended imaging by muscle: (1) For multifidus/erector spinae, use lumbar MRI with multi-echo Dixon (chemical-shift–encoding) for fat fraction (FF), plus T1/T2 for morphology; report CSA, total muscle volume (TMV), and functional muscle volume (FMV). Where available, add T2 mapping (resting T2 as a proxy of contractile quality) and consider diffusion tensor imaging (DTI) for microstructure. Standardize level selection (L4–L5 and L5–S1), slice orientation (true axial to disk space), and region of interest (ROI) depth (superficial vs deep lumbar multifidus) following ISSLS measurement recommendations to improve reproducibility (49, 56, 57). (2) Quadratus lumborum begin with ultrasound (B-mode to define layers; Doppler for hyperemia if suspected enthesopathy; shear-wave elastography for stiffness asymmetry). Ultrasound enables dynamic provocation testing and targeted diagnostic injections; MRI can supplement when broader paraspinal evaluation is already planned, but QL pathology is often functional/myofascial and best captured sonographically (53, 54, 58). (3) Whole-profile biomarkers: For early/occult change, quantitative MRI (qMRI) with Dixon FF is sensitive across lumbar multifidus/ES/PM (and even gluteus medius) and can reveal spatial FI patterns relevant to symptoms and planning rehabilitation. Use harmonized acquisition and segmentation workflows to enable longitudinal tracking and interstudy comparability (59). Suggested practical protocol to ascertain CLBP muscle phenotypes: (1) axial and sagittal T1/T2 from L1–S1; (2) 6-point multi-echo Dixon for FF (calculate per-level lumbar/ES FF, TMV, FMV; report side-to-side asymmetry); (3) T2 mapping if available; (4) optional DTI when researching microstructure; (5) targeted ultrasound with shear-wave elastography of QL when myofascial features or lateral trunk pain are present; (6) adhere to ISSLS measurement standards (slice plane, ROI definition, and depth-specific reporting). This combination captures LM-centric degeneration (primary), ES co-involvement (secondary), and QL myofascial dysfunction (often under-recognized), aligning imaging with the most actionable CLBP muscle phenotypes (56, 57, 59).

The choice of vertebral level is critical for accurately detecting morphological changes such as interfascicular fat infiltration and alterations in the CSA of paraspinal muscles. Evidence from MRI-based studies consistently identifies the lower lumbar segments, particularly L4–L5 and L5–S1, as the most sensitive sites for detecting degenerative and atrophic changes in the multifidus and ES muscles (7, 60). These levels are biomechanically vulnerable due to their higher load-bearing role, increased range of motion, and frequent involvement in degenerative pathologies. As a result, interfascicular fat accumulation an early marker of muscle quality loss is often more pronounced here than at upper lumbar levels (7, 60).

For quantifying CSA, the L4–L5 level is particularly advantageous. It provides consistent anatomical landmarks, reduced partial-volume effects, and high reproducibility in both manual and automated segmentation methods. Moreover, imaging at this level captures both the superficial and deep components of the multifidus and ES, allowing for depth-specific analysis. Standardizing CSA measurements at L4–L5 also facilitates comparability across studies and clinical follow-ups (61, 62).

For early prediction of changes, some studies suggest that L3–L4 can reveal preclinical alterations in muscle morphology especially in asymptomatic individuals at risk of CLBP before overt degenerative changes appear in lower levels. This may be due to the gradual, cephalad spread of fatty infiltration and atrophy, as well as the influence of posture-related loading patterns (62, 63). Therefore, incorporating both L3–L4 (for early detection) and L4–L5/L5–S1 (for advanced change detection) into imaging protocols offers a balanced approach to assess both predictive and established pathological changes in paraspinal muscles (62, 63).

#### Imaging and paraspinal muscle CSA

**FIGURE 1:**
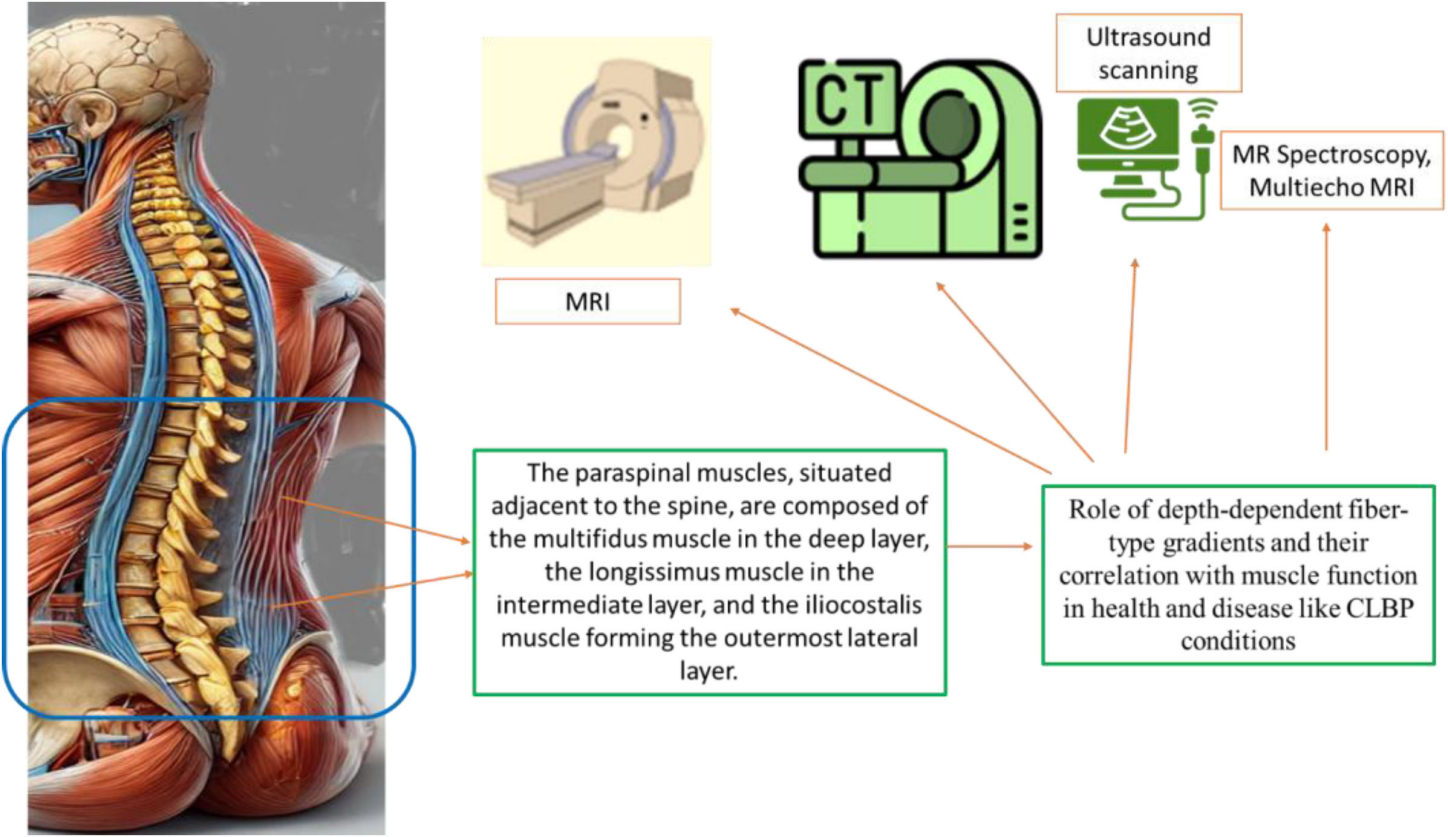
The diverse imaging techniques implicated in the evaluation of paraspinal muscles and their implications in chronic low back pain (CLBP). The current applications of each modality in diagnosing and understanding CLBP, their unique advantages in imaging paraspinal muscle morphology, including cross-sectional area (CSA) and fatty infiltration, and the relevant references supporting these findings. Imaging techniques covered include computed tomography (CT), magnetic resonance imaging (MRI), MR Spectroscopy, Multiecho MRI, and ultrasound (US), emphasizing their specific roles and reliability in the context of CLBP management and research. The techniques need advancements for efficient diagnosis of the CLBP by imaging paraspinal muscle imaging.

Research indicates that reduced CSA of paraspinal muscles correlates with increased LBP and disability (64, 65). While cross-sectional studies extensively examine CSA, morphology, and composition changes due to age, longitudinal data remain limited (64, 66, 67). This describes the need for cohort studies that track long-term muscle dynamics. For instance, structural alterations in the lumbar spine, including changes in CSA, morphology, and muscle composition, have been extensively investigated through cross-sectional research designs (64, 65, 68–71). However, longitudinal studies examining these changes over time remain limited (64, 66, 67). The importance of conducting long-term cohort studies to better understand these dynamics has been emphasized (64). Prior research consistently demonstrates that muscle mass, which diminishes with advancing age, affects various muscle groups, including those in the lumbar region (72, 73). Similarly, the CSA of paraspinal muscles declines with age, particularly in older adults, with men generally exhibiting larger lumbar muscle CSA compared to women (71, 74). Despite these findings, the progression and characteristics of lumbar paraspinal musculature during early adulthood remain poorly understood, as longitudinal population-based studies addressing this developmental stage are scarce (Figure 1).

The precise mechanisms underlying the more pronounced increase in paraspinal CSA among women remain incompletely understood. Muscle dynamics are inherently multifactorial, with muscle size and mass determined by the interplay of protein synthesis and degradation processes, which are further modulated by variables such as nutrition, hormonal fluctuations, injuries, diseases, and physical activity levels (73, 75). For instance, birth weight is shown to have a positive correlation with muscle strength (76) and lean muscle mass in adulthood (77). While relative muscle mass in proportion to body weight starts to decline in 3*^rd^* decade, absolute muscle mass generally remains stable until the fifth decade. This stability is accompanied by a progressive shift in muscle composition, with an increase in intramuscular fat content (12, 78). Consequently, the greater accumulation of intramuscular fat in women may partially explain the observed differences in CSA increments. But it remains unclear whether these changes manifest as early as the third decade of life.

Pregnancy and childbirth might further contribute to these sex-specific differences. Gestational weight gain, a well-documented physiological phenomenon, could potentially lead to alterations in muscle mass and composition (79). In contrast, the observed decrease in CSA of psoas major among men raises distinct questions. Metabolic and endocrinological differences between males and females are well-recognized contributors to variations in muscle dynamics (12, 73, 78). Men tend to experience more pronounced muscle mass losses with aging. However, in women, muscle quality, rather than mass, often deteriorates due to increased fatty infiltration, suggesting divergent mechanisms of muscle degeneration between sexes (13, 78) (Figure 1).

Among both sexes, a previous study (13) findings showed a consistent increase in the CSA of the multifidi bilaterally, with the left multifidus generally larger than the right. Similarly, the erector spinae CSA increased bilaterally, with the left side being more prominent. Conversely, the CSA of the psoas major (PM) exhibited a decline bilaterally in men over the follow-up period. These findings align with earlier reports and suggest that muscle-specific and side-specific dynamics are critical considerations in understanding paraspinal muscle adaptations.

Paraspinal muscle morphology and size have long been implicated in reports examining the etiology of LBP. However, the extent to which lumbar muscle characteristics contribute causally to LBP remains ambiguous (63–65, 80–84). This gap highlights the pressing need for longitudinal studies that can unravel these complex relationships (63, 64). This study provides a valuable perspective by identifying changes in muscle characteristics that could serve as potential predictors or therapeutic targets for LBP, a condition that imposes a significant global disease burden.

As we discussed above, MRI is a significant gold standard to ascertain muscle properties, offering unparalleled detail in assessing CSA, muscle composition, and other morphological features (7, 44–46). Despite its advantages, validation studies comparing MRI with other imaging modalities are limited (7, 45). In this study, measurements were standardized at L4 cranial endplate level, a methodological choice consistent with prior research (71). Emerging technologies, including advanced imaging methods for quantifying muscle fat content (7, 47, 48) and electrodiagnostic techniques (85), may further enhance the assessment of paraspinal muscles.

Fortin et al. (67) performed a longitudinal design, population-based cohort with relatively large sample size, and executed rigorous muscle measurement protocols that examined paraspinal CSA changes in the general population (67) or among patients with LBP (13, 64). According to this report, single-level measurements, while practical, may not fully capture the complex morphological and compositional changes occurring at different spinal levels. Prior studies have criticized this approach and advocated for multilevel assessments to enhance accuracy (86). Nonetheless, this longitudinal dataset allowed for within-subject comparisons, mitigating some limitations associated with single-plane measurements. Further exploration of muscle composition, particularly the role of intramuscular fat and its temporal changes, is necessary to fully understand the observed trends. Thus, dynamic alterations in lumbar paraspinal muscle CSA during early adulthood, revealing sex-specific patterns and their potential implications for musculoskeletal health were elucidated. These findings describe the importance of further longitudinal investigations to elucidate the interplay between muscle morphology, composition, and their roles in LBP development and management (Table 1).

Longitudinal analysis of paraspinal muscle CSA at the L4 cranial endplate in young adults aged 20–30 years revealed sex-specific trends (67). CSA for multifidus and erector spinae enhanced in both sexes, with a more pronounced total muscle area increase observed in women. In contrast, men experienced a decline in psoas major CSA (67). Such variations could stem from hormonal, metabolic, and lifestyle factors, emphasizing the complexity of muscle dynamics (12, 73, 78).

The sex-specific differences in paraspinal muscle CSA changes highlight critical gaps in understanding the interplay between biological and environmental factors. Standardizing procedures to evaluate muscle composition and fat infiltration at various spinal levels (86). Investigating the impact of factors such as birth weight, physical activity, and gestational changes on muscle morphology (76–79). Examining the metabolic and endocrinological pathways underlying sex-specific muscle changes, particularly in relation to aging and LBP development (73, 78). Assessing the reliability of novel imaging modalities, such as multiecho MRI and electrodiagnostics, in diverse clinical settings (7, 13, 47, 48, 85, 86). However, limitations include the reliance on single-level measurements and the absence of detailed muscle composition analysis. Thus, previous reports (13, 64, 67) highlight the potential of advanced imaging techniques to elucidate the structural changes in paraspinal muscles during early adulthood. By addressing existing gaps in research, such as sex-specific muscle dynamics and the role of muscle degeneration in LBP, future studies can contribute to more targeted and effective interventions for managing chronic LBP.

Emerging reports (13, 64, 67) described that imaging technologies provided new opportunities to elucidate the morphological as well as functional features of lumbar paraspinal muscles in CBLP (67). Exploring these variations in fiber type composition and their implications related to muscle function has long been a focus of research. While fiber type analysis is traditionally relies on invasive biopsy techniques, non-invasive imaging techniques now offer efficient reliability to explore the muscle architecture, composition, and function comprehensively. This article describes how these emerging imaging techniques can bridge the knowledge gaps to design future studies.

#### Novel emerging strategies to fiber-type composition analysis

Fiber-type composition in paraspinal muscles can influence spinal stability as well as endurance. Traditional modalities include histochemical staining, could identify alterations inside type I (slow oxidative) and type II (fast glycolytic) fibers in individuals with LBP. Previous reports explored that LBP patients often exhibit a greater proportion of type IIB fibers, associated with mitigated fatigue resistance (87). But, these modalities are reported to be invasive with limited anatomical accuracy and executed at the time of spinal surgeries (87, 88).

Advanced imaging techniques, include qMRI, proton density fat fraction (PDFF) mapping, and ultrasound elastography are now enabling non-invasive evaluation of muscle fiber composition. For example, PDFF can quantify intramuscular fat content that is associated with muscle quality as well as distribution of fiber type. In addition, MRI-based imaging strategies, DTI can provide detailed information related to muscle fiber orientation as well as microstructural integrity that offers insights into functional aspects of fiber-type variations in LBP patients.

A previous report (89) investigated whether increased paraspinal muscle fatigue in individuals with CLBP could be attributed to an altered proportion of type I muscle fibers. The findings demonstrated that although patients with CLBP exhibited significantly reduced muscular strength and endurance compared to healthy controls, no substantial differences were observed in the fiber-type composition of their paraspinal muscles. These results challenge the long-standing hypothesis that an “unfavorable” distribution of muscle fiber types such as a relative deficiency of type I fatigue-resistant fibers predisposes individuals to CLBP. Instead, the evidence suggests that neuromuscular impairments and functional deconditioning, rather than intrinsic fiber-type constitution, may play a more decisive role in the pathophysiology of paraspinal muscle dysfunction in CLBP (89). Mitigated maximum voluntary isometric contraction strength as well as shorter endurance during Sorensen test in CLBP patients elucidate the functional impairments in paraspinal muscles. These results suggest that patients with CLBP exhibit defects in trunk extensor strength and endurance that may contribute to spinal instability and recurrent pain episodes (89). However, the lack of significant differences in surface electromyography (sEMG)-based median frequency declines during fatigue tests between CLBP patients and controls indicates that muscle fatigability is not solely explained by altered electrophysiological responses (89).

Histological analyses (89) revealed comparable proportions and cross-sectional areas of type I and type II muscle fibers between CLBP patients and controls. Type I fibers, characterized by their oxidative metabolism and fatigue resistance, play a critical role in postural support. The absence of significant differences in type I fiber attributes suggests that the structural properties of paraspinal muscles remain intact in CLBP (89). This is consistent with earlier studies that failed to identify substantial histological abnormalities in CLBP populations (89).

Given the lack of evidence linking CLBP to an adverse muscle fiber composition, attention must shift to alternative mechanisms. Potential contributors include neuromuscular control defects, alterations across motor unit recruitment patterns, or altered proprioceptive feedback mechanisms (89). Chronic pain itself may also induce maladaptive changes including muscle atrophy, fatty infiltration, and reduced mitochondrial function, which collectively impair muscle performance. Psychosocial factors, including pain-related fear and kinesiophobia, may further exacerbate motor control dysfunction in these patients (89). The findings emphasize the importance of exploring non-histological factors influencing paraspinal muscle function in CLBP. Advanced imaging techniques, such as magnetic resonance spectroscopy or ultrasound elastography, may provide insights into muscle quality, including fat infiltration and stiffness (89). Additionally, interventions to modulate neuromuscular retraining, proprioceptive enhancement, and psychological rehabilitation should be prioritized in future clinical trials (89). It is crucial to demonstrate the need to adopt a multifaceted strategy to understanding and addressing muscle impairments in CLBP, incorporating biomechanical, neuromuscular, and psychological perspectives (89).

The relationship between paraspinal muscle fiber-type composition as well as CLBP has attained significant attention which has implications for designing rehabilitation strategies. Historically, studies attempting to delineate these differences often overlooked confounding factors such as gender, age, and body size, which can significantly influence muscle morphology (89). Side-specific, muscle-specific, and gender-specific variations in the CSA of paraspinal muscles are well-documented and can be attributed to anatomical, biomechanical, and physiological factors.

Side-specific variations are often linked to handedness, occupational loading, and asymmetric spinal pathology. For example, in individuals with CLBP or lumbar disk herniation, studies (90, 91) have reported a significantly smaller CSA and increased fatty infiltration on the symptomatic side, particularly in the multifidus muscle at the affected segmental level. This asymmetry may be due to disuse atrophy from pain-avoidance postures, denervation from nerve root compression, or compensatory hypertrophy on the contralateral side (90–92). Muscle-specific variations arise from differences in functional roles and fiber-type composition between the paraspinal muscles. The multifidus, located deep and close to the vertebrae, has a higher proportion of type I oxidative fibers, making it more prone to disuse atrophy and fatty infiltration in CLBP. In contrast, the erector spinae and quadratus lumborum are more involved in gross trunk movements and load transfer, often showing less severe but more diffuse changes in CSA. The psoas major, although not always grouped with the “paraspinal” muscles, frequently retains CSA in degenerative conditions, likely due to its hip flexor function and less direct involvement in segmental stability (92–94). Gender-specific variations are largely explained by differences in hormonal milieu, muscle mass distribution, and habitual activity levels. Males typically exhibit a larger absolute CSA across paraspinal muscles due to greater overall lean muscle mass, but when CSA is normalized to vertebral body area or body size, differences between sexes diminish or vary by muscle group. Estrogen has been suggested to influence connective tissue composition and fat infiltration rates, potentially explaining higher fatty infiltration percentages in females in some MRI studies (91, 92, 95, 96). Additionally, pregnancy-related changes in the lumbar spine and trunk musculature may influence CSA in females, particularly in the MF and ES (91, 92, 95, 96). Collectively, these variations highlight the importance of stratifying paraspinal muscle analysis by side, specific muscle, and sex in both research and clinical imaging to ensure accurate interpretation of morphological changes.

For ascertaining the fiber-specific or muscle specific variations, histochemical analyses were conducted on lumbar paraspinal muscle samples obtained during spinal surgery from 21 CLBP patients and via percutaneous biopsy from 21 matched controls (89). Results revealed a notable shift in fiber-type distribution, with CLBP patients exhibiting a higher proportion of type IIB fibers (fast-twitch glycolytic) and mitigated levels of type I fibers (slow oxidative) than healthy individuals, which suggests a glycolytic phenotype in CLBP patients, potentially rendering these muscles more susceptible to fatigue (87).

#### Fiber-type distributions in healthy and pathological states

Determination of the fiber-type distribution in paraspinal muscles has traditionally relied on invasive strategies, including biopsies at the time of spinal surgeries or needle extractions (88). These methods are often anatomically imprecise, relying on proximity to the spinous processes for localization (88). More recently, minimally invasive techniques, such as those using tubular retractors, have improved anatomical precision, enabling detailed comparisons across different muscles and depths. In one study (88), biopsies from the multifidus, longissimus, iliocostalis, and psoas muscles revealed distinct patterns of myosin heavy chain (MyHC) isoform distribution (88). Posterior paraspinal muscles displayed a higher proportion of type I fibers (∼63%), consistent with their postural role, while the psoas muscle exhibited a predominance of type II fibers, aligning with its dual role as a hip flexor and spinal stabilizer (88).

#### Depth-dependent fiber-type gradients

It has been hypothesized that the multifidus muscle’s deeper fibers, which contribute to spinal stabilization, possess a greater proportion of type I fibers than its superficial fibers, which function phasically as spinal rotators and extensors (88). Studies employing histochemical staining for ATPase activity or SDS-PAGE electrophoresis to analyze MyHC distribution found no significant depth-dependent differences in fiber-type composition across multifidus biopsies (88). This suggests that other factors, such as extracellular matrix composition and biomechanical properties, may play a greater role in defining the functional specialization of paraspinal muscle layers (88).

Chronic low back pain patients frequently exhibit histopathological abnormalities in paraspinal muscles, including fiber-type-specific atrophy, fatty infiltration, and core-target fibers. However, evidence remains inconsistent regarding changes in overall fiber-type distribution. Mitigated endurance and greater fatigability in CLBP patients are well-documented, demonstrating that these factors beyond fiber-type distribution including neural activation defects or metabolic inefficiencies, may underlie these impairments (88). Muscle architecture, including fiber length, physiological cross-sectional area (PCSA), and connective tissue composition, significantly influences function. For example, the multifidus muscle’s short fibers and high PCSA enable it to generate large stabilizing forces over short excursions, making it uniquely suited for spinal stabilization (97, 98). Conversely, the psoas muscle, with its longer fibers and lower PCSA, functions more efficiently as a spinal flexor. These architectural differences highlight the significance of considering both intracellular and extracellular components at the time of evaluating muscle function. Future studies should explore how these architectural features adapt in response to CLBP and whether targeted interventions can restore normal function (88). The findings demonstrate the need to move beyond fiber-type distribution as main focus to understand paraspinal muscle dysfunction in CLBP conditions. Hence, it is significant to explore usage of these techniques which could elucidate how muscle adaptations can lead to progression of CLBP to develop novel rehabilitation strategies (87, 99).

In addition, therapeutic strategies that modulate muscle metabolism as well as neuromuscular activation, rather than simply fiber-type composition, may generate good clinical outcomes with higher clinical improvement in CLBP patients (88). For example, resistance training protocols designed to promote oxidative capacity and reduce fatigability in type IIB fibers could mitigate the functional damage observed in these CLBP patients (89, 100–105). In conclusion, while paraspinal muscle dysfunction in CLBP is not directly attributable to adverse fiber-type composition, the interplay of architectural, metabolic, and neural factors warrants further investigation. Integrating advanced imaging, molecular analyses, and biomechanical modeling could generate a more profound understanding of these complex interactions and guide the development of targeted therapeutic approaches (88).

The deep fibers of the multifidus, serving a stabilizing role, are hypothesized to contain more type I fibers than the superficial fibers that function as extensors and rotators (106–108). While minimally invasive biopsy studies have provided some evidence, imaging advancements such as multi-parametric MRI can allow for depth-specific analysis of muscle architecture without requiring surgical access. Such techniques can validate the proposed gradients *in vivo* and investigate their relationship with LBP onset and progression.

Fatty infiltration of paraspinal muscles is a hallmark of LBP and spinal degeneration, potentially altering the functional capacity of these muscles. While earlier studies identified fat infiltration using cadaveric samples or subjective visual grading on imaging (99), advanced imaging modalities now offer objective quantification. For instance, chemical shift encoding-based water-fat imaging can precisely measure fat content and its spatial distribution within the paraspinal muscles. It is necessary to explore how these quantitative measures correlate with clinical outcomes, such as pain severity and functional disability, in LBP patients.

Emerging evidence (88, 97) highlights the importance of extracellular components in determining muscle function, beyond the intracellular fiber composition. Differences in extracellular matrix (ECM) composition, such as collagen density and stiffness, may contribute to the biomechanical properties of paraspinal muscles (97). Elastography, a technique that measures tissue stiffness, can non-invasively assess ECM properties and provide additional data on muscle health and adaptability. Combining elastography with advanced MRI could offer a more comprehensive understanding of muscle function in LBP (88). Prospective, longitudinal imaging studies are essential to assess how muscle adaptations, such as fatty infiltration and fiber-type shifts, contribute to LBP development and progression. Incorporating imaging findings into computational models of spinal biomechanics can help elucidate how muscle changes affect load distribution and spinal stability. Targeted rehabilitation strategies: Imaging data can guide personalized rehabilitation programs by identifying specific muscle deficits, such as reduced type I fiber proportion or increased fat infiltration. Exploration of novel therapies: Future research could explore interventions, such as focused electrical stimulation or regenerative therapies, aimed at restoring muscle quality and function based on imaging biomarkers (88). Advancements in imaging techniques are revolutionizing the study of lumbar paraspinal muscles in chronic LBP. By enabling non-invasive, detailed assessments of muscle morphology, composition, and function, these technologies provide new insights into the pathophysiology of LBP. Integrating these findings into clinical practice will be pivotal for developing targeted therapeutic strategies and improving patient outcomes (88).

### Functional MRI and paraspinal imaging

While traditional imaging modalities like MRI and CT are predominantly implicated to evaluate the CSA of these muscles, their sensitivity in detecting subtle atrophic changes, particularly in CLBP, remains limited (35, 109–112). The inability to differentiate atrophic muscle tissue from fat and fibrous infiltration reduces the diagnostic value of CSA, as such infiltration may occur without significant morphological changes (111, 112). These limitations necessitate imaging methods capable of assessing not just structural changes but also functional and compositional alterations in the paraspinal musculature (112).

Blood oxygen level-dependent (BOLD) MRI has emerged as a promising strategy for assessing microcirculatory dynamics and muscular oxygenation (113–116). By measuring the transverse relaxation rate (R2*), which reflects the deoxyhemoglobin-to-oxyhemoglobin ratio, BOLD MRI provides insights into peripheral muscle perfusion. Complementary to this, T2 mapping quantifies transverse relaxation time and can reveal biochemical changes such as alterations in water metabolism, lactic acid concentration, and fat degeneration (117). These advanced imaging approaches have demonstrated utility in evaluating muscle function and pathology in various conditions, including peripheral arterial disease and muscular dystrophies (118–121). Emerging evidence (122) suggests that combining CSA, BOLD, and T2 mapping could significantly enhance the understanding of paraspinal muscle function in CLBP. For instance, future investigations should assess pre- and post-exercise changes in these parameters, correlating them with muscle endurance, fatigue resistance, and recovery patterns. Longitudinal studies involving healthy participants and patients with CLBP would help delineate the physiological adaptations of paraspinal muscles to rehabilitation protocols. Such studies could also identify potential biomarkers for monitoring treatment efficacy and predicting clinical outcomes (122).

Additionally, BOLD MRI and T2 mapping could facilitate the study of segmental differences in the multifidus, iliocostalis, and longissimus muscles. Investigating these variations may uncover unique patterns of muscle dysfunction in specific spinal regions, contributing to more targeted therapeutic strategies. Advances in artificial intelligence (AI) and machine learning could also aid in analyzing complex imaging datasets, allowing for more precise quantification of muscular changes and their clinical implications (Table 2) (122).

**TABLE 2:** This table emphasizes key advancements, compares efficiencies, and highlights unique benefits of each imaging modality pertinent to parasprinal muscles, providing a comprehensive overview.

Imaging Technique	Advancement to image paraspinal muscle	Efficiency of imaging modality compared to previous	Advantages	References
Laser Doppler flowmetry (LDF)	Measures superficial muscle perfusion but lacks depth penetration, limiting its use for deep lumbar muscle evaluation.	Limited in assessing deeper muscles, as it is invasive and has restricted depth penetration.	Provides accurate superficial perfusion data.	(123–124)
BOLD MRI	Offers non-invasive, high-resolution imaging of microcirculatory perfusion and oxygenation in muscles of varying depths.	Outperforms LDF by combining functional and anatomical data, enabling evaluation of both surface and deep muscle layers.	Provides comprehensive insights into muscle perfusion and oxygenation, suitable for assessing complex lumbar musculature.	(113, 116, 125–127)
T2 mapping	Quantifies changes in water content, osmotic pressure, and lactic acid metabolism to reflect real-time muscle activation and fatigue.	Adds diagnostic precision by highlighting metabolic and biochemical responses of muscles under physical stress.	Valuable for tracking therapeutic intervention effects and monitoring muscle function over time.	(122, 128–130)
IDEAL-IQ imaging	Differentiates between water and fat components within muscles to measure fat fraction (FF) and assess muscle degeneration.	Superior to single-parameter methods by combining fat quantification and structural assessment.	Enhances diagnostic accuracy by providing detailed muscle composition analysis, crucial for evaluating multifidus degeneration in CLBP.	(146–148)
Functional MRI (fMRI)	Captures dynamic muscle activation and functional changes during tasks such as bending or lifting.	Enables real-time imaging of muscle functionality, offering a more dynamic evaluation compared to static modalities.	Useful for studying the biomechanical and functional role of paraspinal muscles during load-bearing activities.	(122, 189)
Diffusion tensor imaging (DTI)	Examines muscle fiber integrity and neuromuscular connectivity, revealing microstructural changes linked to functional impairments.	Emerging as a potential method for detecting subtle alterations not visible on conventional MRI.	Helps to elucidate the interplay between structural and functional muscle changes, enhancing precision in diagnosing muscle degeneration.	(189, 191)
Magnetic resonance spectroscopy (MRS)	Assesses metabolic alterations in paraspinal muscles, such as shifts in biochemical profiles due to degeneration or exercise.	Provides metabolic insights absent in conventional imaging techniques, augmenting the understanding of muscle pathology.	Enables comprehensive evaluation of muscle function at a cellular level, aiding in personalized rehabilitation approaches.	(29, 189)
Dynamic contrast-enhanced mri	Evaluates vascular health and perfusion function in paraspinal muscles, focusing on microvascular changes.	More detailed than static imaging by visualizing dynamic blood flow and oxygenation patterns.	Facilitates assessment of exercise-induced changes in capillary density and vascular adaptations, relevant for CLBP management.	(122, 189)

#### Advances in radiological techniques for paraspinal imaging

While methods like laser Doppler flowmetry (LDF) provide accurate measurements of superficial muscle perfusion, their invasive nature and limited depth penetration restrict their utility for evaluating deep lumbar muscles (123–124). In contrast, BOLD MRI offers non-invasive, high-resolution insights into microcirculatory blood perfusion and oxygenation across various tissue depths (113, 116, 125). By integrating anatomical mapping with functional data, BOLD MRI surpasses LDF in assessing both surface and deep muscle layers, making it particularly suitable for evaluating the complex musculature of the lower back (126, 127) (Table 2). T2 mapping adds another layer of diagnostic capability by quantifying changes in water content, osmotic pressure, and lactic acid metabolism, reflecting real-time muscle activation and fatigue (128–130). Future research could investigate the utility of T2 mapping in monitoring therapeutic interventions, such as exercise programs or pharmacological treatments, by linking T2 changes with clinical improvements in pain and mobility. Additionally, understanding the interplay between T2 and R2* values could provide a more nuanced view of the biochemical and vascular responses of paraspinal muscles to physical stress (122).

Despite the promise of advanced imaging techniques, several questions remain unanswered. For example, the interplay between water metabolism, oxygenation, and muscle composition during and after exercise requires deeper exploration. Variations in R2* and T2 values due to factors such as extracellular water content, capillary density, and fat infiltration must be systematically studied to improve the specificity of these metrics for assessing muscle activation (113, 118, 122). Moreover, the relationship between imaging findings and clinical outcomes in CLBP needs to be clarified. Are changes in BOLD and T2 values predictive of functional improvements, or are they merely indicative of underlying pathology? Addressing such questions will require longitudinal studies combining imaging with biomechanical assessments, electrophysiology, and patient-reported outcomes (122).

To fully harness the potential of advanced imaging in CLBP management, future studies should focus on the following areas such as developing protocols for real-time imaging of muscle activation during functional tasks, such as lifting or bending, to understand the role of paraspinal muscles in load-bearing and movement. Investigate the utility of BOLD MRI and T2 mapping as biomarkers for tracking muscular adaptations to specific rehabilitation interventions, such as core stabilization exercises or electrical stimulation therapies. Explore AI-driven image analysis for identifying subtle patterns of muscle degeneration or recovery that may not be visually apparent in traditional imaging. Conduct head-to-head comparisons of BOLD and T2 mapping with emerging modalities, such as diffusion tensor imaging and elastography, to establish the most effective approach for evaluating paraspinal muscle function. By expanding the scope of research into imaging advancements, clinicians and researchers can gain a more holistic understanding of paraspinal muscle dynamics, ultimately improving diagnostic precision and therapeutic outcomes for patients with CLBP (122). Future research should explore the integration of BOLD MRI and T2 mapping for dynamic, exercise-induced assessments of lumbar paraspinal muscles, enabling a comprehensive evaluation of muscle activation and microcirculatory adaptations (122).

#### Exploring structural and functional changes in paraspinal muscles in CLBP by advanced imaging

**FIGURE 2:**
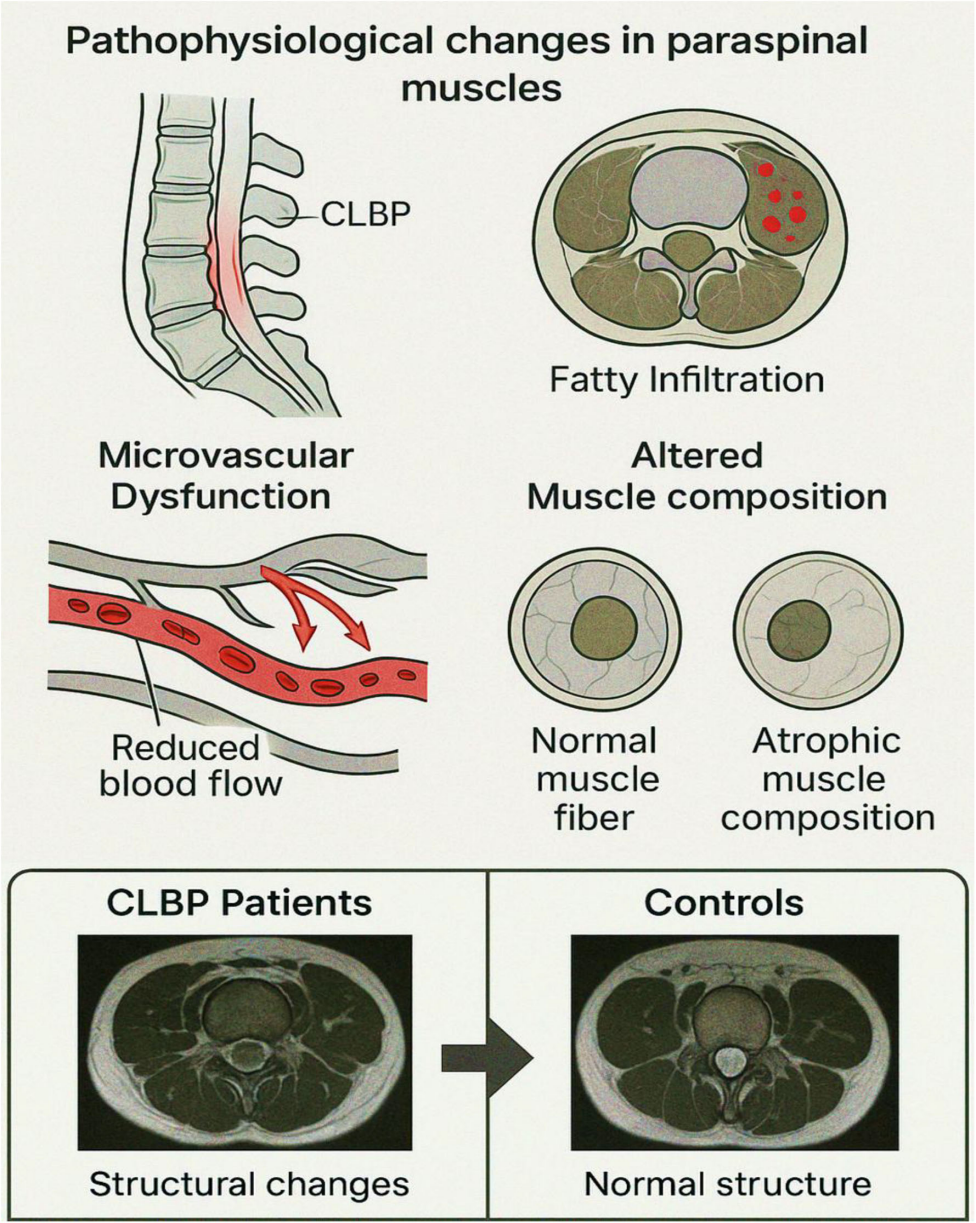
Pathophysiological changes in paraspinal muscles associated with chronic low back pain (CLBP). This schematic illustration highlights key pathophysiological alterations in paraspinal muscles observed in individuals with CLBP: Sagittal view of the lumbar spine depicting the region affected by CLBP, with localized inflammation or dysfunction in the paraspinal musculature. Axial view showing fatty infiltration within paraspinal muscles, a hallmark of chronic degeneration, indicated by red-marked regions. Diagram of microvascular dysfunction illustrating reduced capillary blood flow, a condition contributing to impaired oxygenation and muscle metabolism. Representation of altered muscle composition: normal muscle fiber morphology compared to atrophic muscle fibers commonly seen in chronic muscle disuse or degeneration. These cumulative structural and microvascular alterations underlie the functional impairments and pain associated with CLBP. This figure illustrates the interplay between structural degeneration and functional impairments of paraspinal muscles in individuals with CLBP. Aanatomy and imaging sites: sagittal and axial schematic views of the lumbar spine, highlighting the L4/5 and L5/S1 intervertebral disk levels. Paraspinal muscles (multifidus and erector spinae) are labeled. (BOLD imaging: Functional heatmaps derived from BOLD MRI show elevated R2* values (red-yellow areas) in CLBP patients compared to controls, particularly at L5/S1, suggesting impaired oxygenation and microvascular dysfunction.

Chronic low back pain has been associated with notable structural alterations in the paraspinal muscles; but whether these alterations extend to functional impairments remains less understood. This study investigated metabolic and perfusion function changes in paraspinal muscles among patients diagnosed with CLBP, as inferred through BOLD imaging and T2 mapping (Figure 2). By leveraging these advanced imaging modalities, the study by Chen et al. (131) ascertained the interplay between structural and functional characteristics of the lumbar paraspinal musculature in CLBP (131). Participants in this study (131) were consecutively recruited from December 2019 to November 2020, including CLBP patients and asymptomatic controls. Diagnosis of CLBP was confirmed in outpatient settings, while control participants exhibited no CLBP or related disorders. Functional MRI (fMRI) techniques, including BOLD imaging as well as T2 mapping, were performed at L4-S1 intervertebral disc levels. Measurements of effective transverse relaxation rate (R2* values) and transverse relaxation time (T2 values) were taken at the central planes of the L4/5 and L5/S1 discs (131).

The results (131) revealed significantly higher R2* values in paraspinal muscles of CLBP patients compared to controls, indicative of impaired metabolic and perfusion functionality. This finding aligns with the physiological principles underpinning BOLD imaging, where R2* values correlate with tissue deoxyhemoglobin levels (116, 132–134). Elevated deoxyhemoglobin concentration suggests reduced oxygenation and microvascular dysfunction in paraspinal muscles. Supporting this hypothesis, prior studies have demonstrated diminished vascular density in the lumbar multifidus muscle in individuals with chronic degenerative lumbar conditions (135). Interestingly, the R2* values were more pronounced at the L5/S1 level compared to L4/5, potentially reflecting greater fatty infiltration in the lower lumbar region. This observation elucidates the need for further exploration of segmental variations in perfusion and metabolism across lumbar levels in CLBP patients.

#### Challenges in T2 mapping interpretation

Contrary to the R2* findings, T2 values in CLBP patients were shorter than those in controls, though not significantly. T2 values are influenced by tissue water content, with higher water levels typically associated with prolonged T2 times (136, 137). However, fatty infiltration, a hallmark of paraspinal muscle degeneration in CLBP can confound T2 measurements by altering water-fat composition (33, 138–140). The coexistence of water and fat within muscle complicates the differentiation of their contributions to T2 values. Advanced techniques like the IDEAL–Carr-Purcell-Meiboom-Gill (CPMG) pulse sequence could isolate water-specific T2 values, offering clearer insights into muscle tissue composition (141).

#### Implications of age-related microvascular changes in paraspinal muscles and imaging

A previous study (142) also observed a positive correlation between R2* values and age in both groups, suggesting age-related declines in microvascular function. Previous research has documented reduced capillary density and impaired microcirculation with aging, further supporting these findings (142). However, the dynamic interplay between water metabolism, oxygenation, and biological factors such as hemoglobin levels and extracellular water content warrants further investigation to elucidate age-related changes in paraspinal muscle perfusion. Asymmetry in paraspinal muscle structure, often reported in CLBP, may contribute to discrepancies in functional imaging parameters (143, 144). While this study did not identify significant differences in fMRI parameters based on structural asymmetry, the lack of dominant limb assessment and potential asynchronous structural-functional changes may have influenced the results. Further reports should describe these variables to efficiently ascertain their influence on muscle metabolism and perfusion.

Given the observed dysfunction in muscle perfusion, targeted rehabilitation strategies focusing on moderate exercise may enhance microvascular function and metabolic capacity in CLBP patients. Exercise-induced increases in capillary density and improved perfusion function have been reported in previous studies, describing the therapeutic potential of such interventions (145). However, much of the available evidence is derived from studies with relatively small sample sizes and methodological constraints. For example, several investigations excluded important confounding factors such as circulating lactate concentrations, extracellular water content, and systemic metabolic influences, all of which can substantially affect perfusion and muscle metabolism outcomes. These limitations restrict the generalizability of the findings. Future research should therefore focus on larger, longitudinal studies that integrate advanced imaging modalities (e.g., BOLD MRI and T2 mapping) with comprehensive biochemical and physiological assessments. Correlating imaging-derived biomarkers with clinical outcomes over time would not only strengthen causal inferences but also clarify whether exercise-driven microvascular improvements translate into durable pain relief and functional recovery in CLBP.

For instance, a previous report provided foundational evidence for the utility of advanced imaging techniques like BOLD and T2 mapping in assessing paraspinal muscle function (131). Incorporating advanced imaging modalities may generate a more holistic understanding of metabolic and perfusion dynamics in CLBP (131). Future studies should employ larger, more diverse populations and explore the integration of novel imaging techniques including diffusion-weighted imaging and elastography, to further enhance the evaluation of paraspinal muscle function in CLBP (131). By addressing the outlined gaps, it is crucial to refine these methodologies, paving the way for improved diagnostics and personalized therapeutic approaches in CLBP management (131).

### qMRI advances for imaging of paraspinal muscles in CLBP

Quantitative MRI offers significant potential for advancing the evaluation of paraspinal muscles in CLBP patients. A previous study (146) ascertained the implications of qMRI in analyzing young individuals with chronic non-specific low back pain (CNLBP) and unilateral symptoms. Utilizing advanced imaging strategies such as T2 mapping and IDEAL-IQ scans, previous studies (146) identified distinct differences in T2 values and fat fraction (FF) between symptomatic and asymptomatic sides of the multifidus and erector spinae muscles at various lumbar levels. These findings conclude the importance of combining T2 and FF metrics to enhance diagnostic accuracy, as demonstrated by their combined model yielding an AUC of 0.91 for distinguishing affected individuals (Table 2) (146).

The multifidus and erector spinae muscles are integral to spinal stability and motion control, and their pathology can disrupt core musculature function. T2 values (147, 148), which reflect changes in water content, inflammation, and fibrotic degeneration, were elevated in pain-side muscles at L5, indicating significant microstructural changes preceding visible morphological alterations. This suggests that T2 mapping could serve as a sensitive biomarker for early detection of muscle pathology in CNLBP patients. Fat infiltration, a hallmark of muscle degeneration, is quantified as FF via IDEAL-IQ imaging (147, 148). Higher FF in symptomatic muscles suggests that abnormal postures or movement patterns may exacerbate degeneration, particularly in the multifidus, which contributes significantly to spinal dynamic stability (149–151). These findings highlight the potential of FF as a complementary marker to T2 values, providing a more comprehensive assessment of paraspinal muscle pathology in CNLBP.

#### Recent developments in MRI Dixon technology and paraspinal muscles

Dixon method is an MRI technique designed to differentiate between water and fat signals, thereby enhancing image clarity and diagnostic accuracy (152, 153). Advancements in MRI Dixon technology have significantly enhanced the assessment of paraspinal muscles, offering precise quantification of muscle composition and aiding in the diagnosis and management of various spinal conditions (154, 155). (1) Two-point Dixon method (LAVA-FLEX) is a technique that has shown high reproducibility in assessing FFs of lumbar vertebral bodies and paraspinal muscles. However, it tends to slightly overestimate FFs compared to magnetic resonance spectroscopy (154–156). Six-point Dixon method (IDEAL-IQ) is a method that exhibits higher correlation and better agreement with MRS-derived FFs than two-point Dixon method, making it a more accurate alternative for muscle composition analysis (154–157). The application of Dixon MRI techniques in assessing paraspinal muscles has led to several clinical insights. For example, fatty infiltration and LBP: Increased fatty infiltration in paraspinal muscles, particularly the multifidus and erector spinae, has been associated with chronic LBP (154–157). Quantitative MRI assessments have revealed higher mean signal intensities in these muscles on the painful side, indicating greater fat content.

#### Quantitative imaging for early detection

Advanced MRI techniques, including chemical shift encoding-based water-fat MRI (CSE-MRI) (130–133), enable objective and quantitative assessment of muscle composition. These methods facilitate early detection of muscle degeneration, allowing for timely intervention and potentially improved patient outcomes. Integrating advanced MRI Dixon techniques into clinical practice enhances the evaluation of paraspinal muscles by providing accurate measurements of muscle composition and structural changes. These advancements contribute to a better understanding of the relationship between muscle health and spinal disorders, ultimately informing more effective diagnosis, treatment planning, and monitoring of therapeutic interventions. Integrating MRI Dixon technology into the assessment of paraspinal muscles represents a significant advancement in medical imaging, particularly when combined with CT scans. Initially limited by technological constraints, the method has undergone substantial refinements over the past two decades, leading to its widespread adoption in clinical practice (152–157).

Recent developments in MRI technology have further enhanced the capabilities of the Dixon method. For example, the incorporation of AI into MRI scans has increased precision, enabled more detailed scan results and can facilitate more accurate diagnoses. High-resolution imaging can be achieved using MRI Dixon technology. Advancements in MRI technology have significantly improved image quality, allowing for more detailed visualization of soft tissues (152–157). Assessing the condition of paraspinal muscles is vital in diagnosing and managing various spinal disorders. Traditional imaging techniques like CT and standard MRI provide valuable information but have limitations in distinguishing between fat and water content within muscles (152–157). Dixon method addresses this by offering a clear separation of fat and water signals, leading to more accurate assessments of muscle composition (152–157). As we discussed above, the implementation of Dixon method in evaluating paraspinal muscles can lead to a higher diagnostic accuracy by precisely differentiating between fat infiltration and muscle tissue, clinicians can better diagnose conditions such as muscle atrophy or degeneration (152–157). These techniques enable improved treatment planning through which accurate imaging informs more effective rehabilitation strategies and surgical interventions by providing detailed insights into muscle health. In addition, these techniques also enable comprehensive monitoring thereby the ability to monitor changes in muscle composition over time allows for the assessment of disease progression and the effectiveness of therapeutic modalities (152–157). Incorporating MRI Dixon technology into any future research project focusing on CT, MRI, and paraspinal muscles is a forward-thinking approach. This integration not only enhances the precision of muscle assessments but also aligns with the latest advancements in medical imaging, potentially leading to improved patient outcomes (152–157).

#### Advancements in MRI techniques for lumbar paraspinal muscle fat fraction assessment

Focusing on the fat fraction of lumbar paraspinal muscles has emerged as a major research area, offering deeper insights into spinal health and associated pathologies. Recent advancements in MRI techniques have significantly enhanced the accuracy and reliability of FF assessments, particularly in the context of lumbar paraspinal muscles (152–158). CSE-MRI, particularly the Dixon method, has emerged as a robust technique for quantifying the PDFF in muscles (152–157). This method offers a more accurate assessment of fat infiltration than traditional imaging sequences. Quantification of fat infiltration in lumbar paraspinal muscles has significant clinical implications (1) CLBP: a multicenter prospective study involving 493 patients with CLBP demonstrated a correlation between increased fat infiltration in the multifidus and erector spinae muscles and the severity of lumbar spine pathologies (159). The study involved the usage of qMRI to measure CSA and PDFF of these muscles, highlighting the importance of accurate fat quantification in understanding CLBP (152–157). (2) lumbar spinal stenosis (LSS): In patients with LSS, fatty infiltration of the paraspinal muscles has been associated with pain and disability (152–157). A novel muscle fat index demonstrated high observer reliability in assessing fatty infiltration using routine lumbar MRI examinations, elucidating the potential of FF measurements in clinical evaluations (152–157). Comparison of imaging modalities: Research comparing the IDEAL (Iterative Decomposition of water and fat with Echo Asymmetry and Least-squares estimation) sequence with traditional T2-weighted MRI for muscle composition measurements revealed that the IDEAL sequence provides more accurate assessments of fat infiltration (160). This finding suggests that incorporating advanced imaging sequences can enhance the precision of FF measurements (160). Texture analysis: the application of texture analysis to PDFF maps derived from CSE-MRI has shown promise in serving as a surrogate marker for tissue structure (152–157, 161). This approach could potentially improve the prediction of paraspinal muscle strength beyond muscle volume assessments, offering a more comprehensive understanding of muscle health (152–157, 161). In conclusion, shifting the research focus to the fat fraction of lumbar paraspinal muscles, facilitated by advancements in MRI technology, offers a more nuanced understanding of spinal health. Accurate quantification of intramuscular fat is crucial for diagnosing, monitoring, and developing targeted interventions for various spinal conditions. Continued research in this area is essential to fully harness the potential of these imaging advancements in clinical practice (152–157, 161).

Computed tomography imaging has been instrumental in evaluating the morphology and pathology of paraspinal muscles. Recent studies have focused on the following advancements related to the CT scan (152–157, 161). For example, quantitative assessment: CT scans facilitate precise measurement of CSA and density of paraspinal muscles. A study (162) involving 31 patients assessed these parameters at different spinal levels, demonstrating the utility of CT in evaluating muscle degeneration and its correlation with spinal pathologies (162). Fatty degeneration analysis: CT imaging has been utilized to detect fatty infiltration in paraspinal muscles, which is associated with conditions like CLBP. Research (163, 164) indicates that increased fat content within these muscles correlates with higher pain levels and functional impairment. Automated segmentation: these advancements in machine learning have led to the development of automated segmentation techniques for paraspinal muscles in CT scans (163, 164). Implementing U-Net-based architectures has improved the accuracy of body composition analyses, aiding in the early detection of muscle degeneration (152–157, 161, 163).

Magnetic resonance imaging offers superior soft-tissue contrast, making it invaluable for detailed assessment of paraspinal muscles to ascertain paraspinal muscle atrophy. Recent advancements include: (1) A study assessing the relationship between MRI findings and paraspinal muscle morphology in CLBP patients highlighted the significance of fat infiltration in understanding muscle degeneration (152–157, 161). Previous reports describe that atrophy of these muscles occurs in 20%–60% of people suffering from chronic LBP, elucidating the importance of MRI in early diagnosis and management (152–157, 161). Additionally, automated analysis of images includes development of population-averaged MRI atlases, which have facilitated automated image analysis of paraspinal muscles. These tools enhance the efficiency and accuracy of muscle assessments, aiding in large-scale studies and clinical evaluations (152–157, 161).

Furthermore, the integration of advanced CT and MRI techniques in assessing paraspinal muscles has several clinical implications. Reliable imaging techniques enables early detection of muscle degeneration, timely interventions, and individualized rehabilitation or surgical planning, thereby improving patient outcomes (152–157, 161). Recent advancements in CT and MRI have further enhanced the evaluation of paraspinal muscles, advancing diagnosis, treatment, and understanding of spinal disorders (152–157, 161).

#### X-Ray imaging and paraspinal muscles

Recent developments in X-ray imaging have provided deeper insights into muscle morphology and pathology of paraspinal muscles for reliable diagnosis the management of spinal disorders (165, 166). While X-ray imaging has traditionally been limited in assessing soft tissues like muscles due to its low contrast resolution, recent advancements have expanded its utility for ascertaining paraspinal muscles. For instance, (1) dynamic radiography: Innovations in dynamic X-ray imaging allow for the assessment of functional aspects of the spine and associated musculature (164–167). By capturing real-time movement, clinicians can evaluate muscle function and detect abnormalities that static images might miss. (2) Digital tomosynthesis: This technique provides layered imaging, enhancing the visualization of paraspinal muscles by reducing overlapping structures. It offers a cost-effective alternative to CT scans with lower radiation exposure (165, 166). (3) Dual-energy X-ray absorptiometry (DEXA): Primarily used for bone density measurement, DEXA has been adapted to assess body composition, including muscle mass. This adaptation aids in evaluating muscle degeneration and fat infiltration in paraspinal muscles (152–157, 161, 163–167). A study demonstrated that CT muscle assessment might act as a biomarker for various medical and surgical conditions (152–157, 161, 163–167). Automated segmentation: Advancements in machine learning have led to automated segmentation techniques in CT imaging, enhancing the accuracy and efficiency of paraspinal muscle evaluations. A systematic review highlighted the potential of these techniques in clinical settings (152–157, 161, 163–167). It has been applied to various organs, including skeletal muscle, providing quantitative data on muscle elasticity. The integration of deep learning techniques in MRI reconstruction has enhanced image quality and reduced acquisition times. These advancements facilitate more accurate assessments of paraspinal muscles, particularly in challenging scenarios like low-dose imaging (152–157, 161, 163–167). Recent advancements in X-ray imaging, CT, and MRI have significantly improved the assessment of paraspinal muscles. These developments enable more accurate diagnoses and inform targeted therapeutic interventions, enhancing patient outcomes in spinal disorder management (152–157, 161, 163–167, 168–184).

#### Integration of imaging biomarkers for clinical applications

Combining T2 and FF metrics enhances sensitivity and specificity for diagnosing CNLBP, especially at the L5 level. This combination provides insights into both biochemical and structural changes, enabling clinicians to assess the severity of muscle dysfunction more accurately (185). Given the multifidus’ susceptibility to fat infiltration and atrophy, future research should explore advanced imaging techniques like DTI or elastography to elucidate the interplay between structural and functional changes in lumbar muscles.

Additionally, correlations between T2 and FF with clinical scores like the Japanese Orthopedic Association (JOA) and Visual Analogue Scale (VAS) scores suggest that these metrics could serve as non-invasive proxies for functional impairment and pain levels. But these relationships are constrained by narrow age ranges and small sample sizes. Expanding studies to include diverse populations and longitudinal designs could provide more robust evidence for these correlations (64, 137, 146, 186–188).

Investigating natural variations in paraspinal musculature over time offers valuable insights into the progression of CLBP. A recent study tracked changes in muscle morphology and composition over four months, highlighting the significance of asymmetry in cross-sectional area as a potential indicator of pathology (189). Such findings demonstrate the need for longer-term studies that incorporate advanced imaging modalities to capture subtle, time-dependent changes in muscle structure and function. Future research should also address the role of muscle microcirculation and perfusion in CLBP. Strategies include BOLD imaging and dynamic contrast-enhanced MRI could generate deeper insights into the vascular health of paraspinal muscles, which are often compromised in CLBP (189). However, the interplay between fatty infiltration, fibrosis, and muscle atrophy warrants further investigation using advanced imaging sequences capable of isolating these factors (189). Emerging technologies like magnetic resonance spectroscopy could provide detailed metabolic profiles related to the paraspinal muscles, which offer a novel dimension to muscle pathology research. Integrating imaging data with machine learning algorithms could enhance predictive modeling for CLBP progression and treatment outcomes. Such approaches can identify patient-specific patterns of muscle degeneration and guide personalized rehabilitation strategies (189). In conclusion, advances in imaging techniques hold immense promise for transforming the evaluation of lumbar paraspinal muscles in CLBP (189).

#### Implications of paraspinal muscle degeneration and imaging in CLBP

Some studies challenge the significance of paraspinal muscle degeneration in LBP pathogenesis. Another report (60) described the limited predictive value of paraspinal muscle morphology in LBP outcomes. Similarly, another report (190) found no significant differences in multifidus CSA between LBP patients and healthy controls, while Cuellar et al. (43) observed no correlation between muscle size and LBP in older adults. These discrepancies call for refined imaging techniques to uncover subtler pathological features.

Fat infiltration in the multifidus emerges as a more reliable indicator of pain and disability risk, as shown in recent MRI studies (36, 63). Unlike CSA reductions, fatty infiltration alters neuromuscular function and may precede muscle atrophy. Muscle histology and fiber-type alterations: Degenerative changes in paraspinal muscles extend beyond morphology, affecting muscle fiber composition. Healthy paraspinal muscles predominantly contain fatigue-resistant Type I fibers, critical for maintaining spinal stability (192). In CLBP patients, fiber composition shifts toward Type II fibers, accompanied by non-specific abnormalities and Type II fiber atrophy (193, 194). These changes, independent of symptom duration, suggest a long-term impact of CLBP on muscle functionality (87, 195).

Notably, women may exhibit a higher percentage of Type I fibers, potentially conferring greater resilience to CLBP-induced changes (36). Additionally, integrating imaging data with histological findings could elucidate the mechanistic links between fiber-type transformations and pain syndromes. Reversing paraspinal muscle degeneration remains a critical area for future research. Exercise interventions have shown promise in mitigating atrophy and enhancing muscle strength. For instance, stabilization exercises increased multifidus and psoas CSA and reduced pain in patients with degenerative disk disease (196). Similarly, elite athletes with LBP demonstrated improved multifidus symmetry and CSA following a staged stabilization-training program (197).

Randomized controlled trials (RCTs) further validate the efficacy of intensive exercise protocols. A previous RCT (110), biweekly exercise over 15 weeks improved back extensor strength and muscle density, though the changes in CSA were modest (110). Another RCT comparing lumbar fusion and cognitive intervention exercise groups found superior muscle strength in the exercise cohort, emphasizing the benefits of dynamic physical activity (191). Future research should explore optimal exercise regimens tailored to patient-specific muscle degeneration profiles, utilizing real-time imaging feedback for precision training (29). To address current knowledge gaps, advanced imaging technologies must be employed to capture dynamic and structural changes in paraspinal muscles. DTI and functional MRI could elucidate muscle fiber integrity and neuromuscular control, while magnetic resonance spectroscopy could assess metabolic alterations. Combining these modalities with machine learning algorithms may facilitate the development of predictive models for CLBP progression and treatment response. High-resolution imaging over extended periods may reveal critical thresholds where muscle degeneration becomes clinically significant, enabling earlier interventions.

The interplay between paraspinal muscle degeneration and CLBP remains multifaceted, and needs innovative imaging strategies. By leveraging advancements in MRI technology, integrating imaging biomarkers with clinical data, and tailoring exercise-based interventions, future research can pave the way for personalized management of CLBP. Addressing the reversibility of muscle degeneration and uncovering its underlying mechanisms will be pivotal in reducing the burden of CLBP and improving patient outcomes (29).

## Conclusion

Emerging advances pertinent to the imaging techniques have typically transformed our understanding of paraspinal muscle morphology and function in CLBP. Modern modalities such as fMRI, qMRI, and T2 mapping have enabled the detailed visualization of structural and functional changes in these muscles, shedding light on their role in CLBP pathophysiology. Findings related to fiber-type composition, depth-dependent gradients, and microvascular alterations have elucidated the complexity of paraspinal muscle involvement in CLBP. However, the lack of standardized imaging protocols and the challenges in interpreting certain imaging biomarkers, such as T2 mapping data, present significant barriers to widespread clinical implementation. These advancements hold promise for early diagnosis, tailored interventions, and better prognostic evaluations for patients with CLBP.

### Future directions

Advancing the field requires the development of standardized imaging protocols to ensure reproducibility across research and clinical practice. Integrating imaging biomarkers such as fiber-type composition and microvascular metrics with clinical assessments could improve diagnostic and therapeutic precision. Greater emphasis should be placed on depth-specific muscle analyses and the interplay between structural changes (atrophy, fatty infiltration) and functional impairments (neuromuscular control). Emerging technologies, including high-resolution MRI, elastography, and AI-driven image analysis, hold promise for detecting subtle alterations, automating measurements, and predicting outcomes. Longitudinal studies are essential to establish causal links between paraspinal muscle alterations and CLBP progression, ultimately enabling personalized rehabilitation strategies that target specific imaging-identified impairments.
